# From bladder to brain: How you know when it’s time to go

**DOI:** 10.1016/j.conb.2025.103114

**Published:** 2025-10-10

**Authors:** Anne M. J. Verstegen, Kara L. Marshall

**Affiliations:** 1 Division of Nephrology, Department of Medicine, Beth Israel Deaconess Medical Center, Harvard Medical School, Boston, MA, 02215, USA; 2 Department of Neuroscience, Baylor College of Medicine, Houston, TX, 77030, USA; 3 Jan and Dan Duncan Neurological Research Institute at Texas Children’s Hospital, Houston, TX 77030, USA; 4 Howard Hughes Medical Institute, Baylor College of Medicine, Houston, TX, 77030, USA

## Abstract

The decision to urinate relies on assessing bladder fullness and context to determine an appropriate time and place to go. Any disruption in this interoceptive process results in frequent and sometimes debilitating consequences in daily life. Recent work has uncovered key pathways and brain regions that contribute to the sense of bladder stretch and the control of urinary reflexes, but many open questions remain. Here, we review the known mechanisms that convey sensory information from the bladder to the brain and back down again, and we highlight the knowledge gaps and opportunities for better understanding this system, which will be critical to develop effective therapies for urinary dysfunction.

## Introduction

‘*Do you have to go to the bathroom?*’ Most of us remember this question from when we were young, asked right before getting into the car. How can we estimate that our bladder is going to be filled to capacity in the foreseeable future? We have the remarkable ability to assess bladder fullness and control urination through interoceptive sensation from the lower urinary tract (LUT). Here we describe the interoceptive pathways and neural processes that are involved in detecting bladder stretch sensations that indicate it is time to go.

The bladder has two states: filling and voiding (urination). The urinary system relies primarily on mechanical cues that convey fullness to determine when to switch from one state to the other. As the bladder fills, distension is sensed by mechanosensory afferents in the bladder wall that relay this signal to the brain. Barrington’s nucleus in the brainstem is the ultimate gate that initiates urination. The LUT is distinct from some other interoceptive systems in that reflexes drive it but they are not determinative: we have control over when and where voiding occurs. Healthy people experience the sensation of needing to empty their bladder well before the bladder is full, and this allows for preparedness and anticipation. Animals that use urine to mark territory or advertise sexual availability can assess environmental conditions for safety and appropriateness before deciding when to void. Thus, the LUT operates as a reflexive system with straightforward sensory inputs that are also under cognitive control, making it an excellent system to study sensory integration of interoceptive cues and top-down control over physiology.

### Peripheral inputs that mediate stretch detection

**Figure 1:**
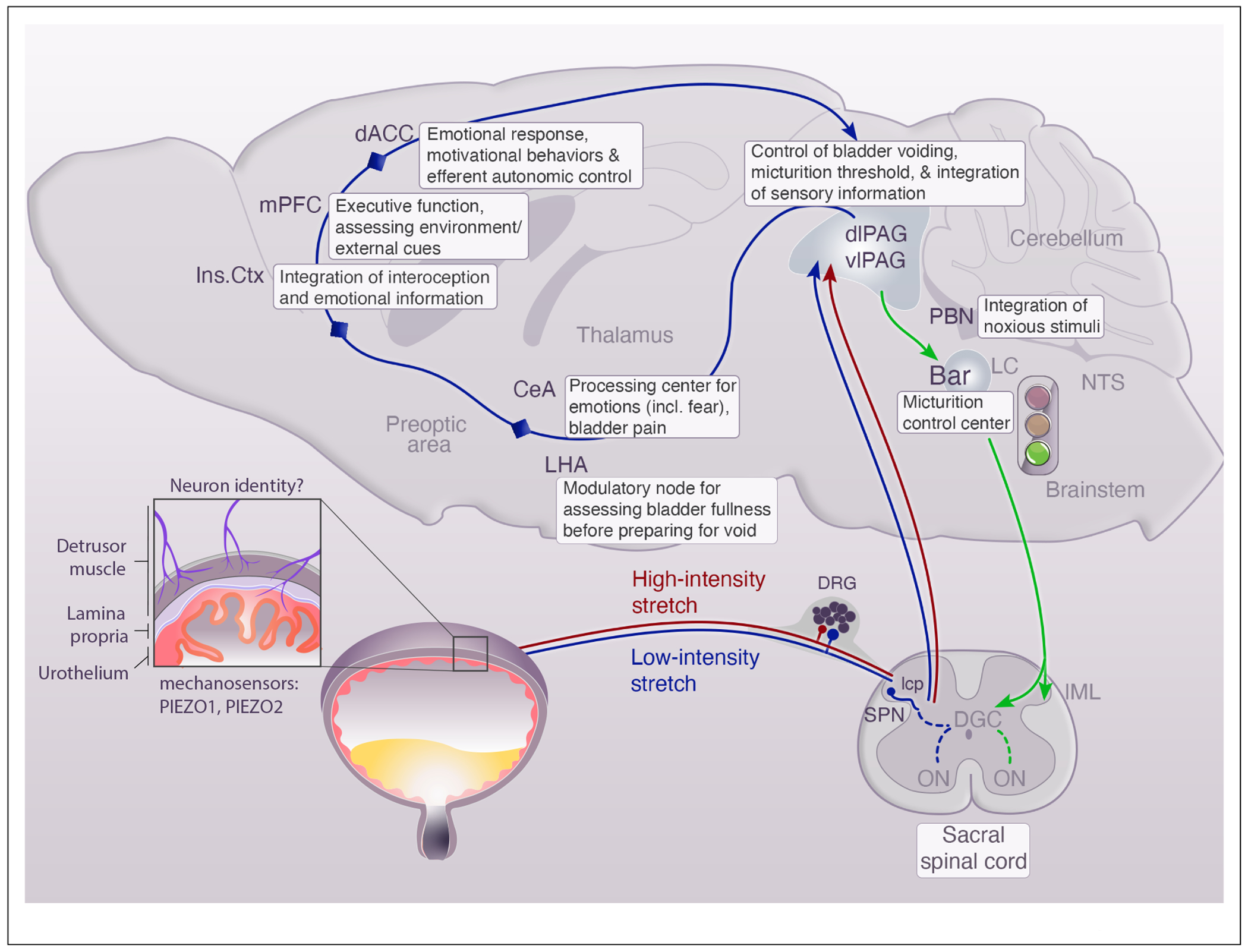
An overview of pathways involved in urinary sensation. Lines indicate general pathways involved in taking information from the lower urinary tract (LUT) through the nervous system. Hypothesized regions encoding low-intensity or innocuous mechanosensory inputs from filling are represented in blue, and hypothesized high-intensity or nociceptive sensory information is shown in red. In the brain, LUT bladder filling information travels through multiple regions that process these cues, conveyed by the blue line without specific anatomical connections drawn. Boxes denote identified functions of brain regions in processing lower urinary tract sensory information and in shaping urinary behaviors. Efferent pathways that drive urination are shown in green. Bar, Barrington’s nucleus; CeA, central nucleus of amygdala; dACC, dorsal anterior cingulate cortex; DGC, dorsal gray commissure; dlPAG, dorsolateral periaqueductal gray; DRG, dorsal root ganglion; Ins.Ctx, insular cortex, IML, intermediolateral cell column; lcp, lateral collateral pathway; LC, locus coeruleus; LHA, lateral hypothalamic area; mPFC, medial prefrontal cortex; NTS, nucleus of the solitary tract; ON, Onuf’s nucleus (dorsolateral nucleus is the rodent equivalent of Onuf’s nucleus); PBN, parabrachial nucleus; PGN, pre-ganglionic neurons; SPN, sacral parasympathetic nucleus; vlPAG, ventrolateral periaqueductal gray.

Sensory neuron signaling is the primary mechanism by which the nervous system monitors bladder fullness. Neurons innervating the bladder are directly activated by stretch as it fills, but they do not work in isolation: mechanical responses from other tissues also contribute to muscle activity and stretch sensation (Figure 1).

#### Non-neuronal contributions to stretch sensation

The epithelial layer facing the bladder lumen, composed of urothelial cells, serves not only as a dynamic barrier but also actively signals to surrounding tissues to shape physiological responses. Urothelial cells express the mechanosensory ion channels PIEZO1, PIEZO2, and TMEM63B [1–3]. Stretching these cells promotes the release of signaling molecules including adenosine triphosphate (ATP), acetylcholine, adenosine, and nitric oxide. The most prominently studied molecule in the context of urothelial signaling is ATP, the release of which depends on PIEZO ion channels[4]. Recent work [5–7] indicates that ATP contributes to urination during inflammation or pathology, rather than driving normal urination. Acetylcholine (ACh) is also released from urothelial cells downstream of PIEZO1-dependent activity [8]. Beneath the urothelium, interstitial cells of the lamina propria are proposed to be mechanically sensitive and to interact with urothelial cells and shape muscle activity [9]. Altogether, signaling molecules released in response to stretch affect sensory neuron activity and muscle tone, which contribute to fullness sensing.

The bladder detrusor muscle itself is not just relaxing during filling; local, asynchronous contraction events occur within muscle fibers due to signaling from urothelial cells, as well as intrinsic myogenic activity and autonomic input [10–12]. These microcontractions are distinct from the coordinated contractions mediated by the parasympathetic nervous system during urination. By enhancing activity in mechanosensory afferents, the microcontractions are thought to contribute to the feeling of fullness [13]. Pathological hyperactivity of the detrusor muscle likely underlies overactive bladder (OAB). In fact, because urgency occurs during filling, the microcontractions influenced by ACh release from the urothelium might be the relevant site of action for antimuscarinic medications used to treat OAB. Although stretch-induced signaling by non-neuronal cells is established, more clarification is needed to determine how bladder muscle activity is modulated in healthy and diseased states, and to understanding how this influences sensory neuron activity.

#### Sensory neuron subtypes and functions

The LUT is unusual for an interoceptive system because it receives spinal innervation from the dorsal root ganglia, and not vagal innervation, although studies have found anatomical [14] and functional evidence for vagal innervation of the bladder in specific species, or after injury in humans [15]. Sensory neurons innervate the LUT via the hypogastric (extending projections from thoracolumbar regions, T12-L2), pelvic, and pudendal nerves (extending from lumbosacral regions, L5-S2). Within the dorsal root ganglia (DRG), neuron subtypes are intermingled, and the LUT receives innervation from medium-to small-diameter A-delta and C-fibers [16]. Both subtypes respond to mechanical force, and mechanoreceptors are found in all bladder layers [17,18] (Figure 1). Although traditional classifications define C-fibers as nociceptive, a variety of mechanosensory neuron types contribute to bladder function within these broad classifications, and more nuanced work is needed to tease apart their functions.

Defining specific sensory neuron functions in the LUT will require linking molecular markers to different cell types and responses. The molecular diversity of functionally defined sensory neuron subtypes has recently been mapped [19–21]. LUT-specific innervating neurons are beginning to be defined using labeling techniques and intersectional genetics to distinguish afferent innervation (from DRGs) from autonomic efferent innervation [22–24]. Retrograde labeling from the bladder has determined that over 75% of bladder afferents are peptidergic and express calcitonin gene-related peptide (CGRP). Approximately 25% express tyrosine hydroxylase (TH) [23], and CGRP-negative cells, likely mechanoreceptors, are TrkB-positive [24]. Studying TH-expressing neurons has been confounded by overlap with expression in the sympathetic nervous system, and the fact that DRGs are not immunoreactive for TH in the periphery. In the skin, TH + neurons are C-low-threshold touch receptors, but their role in the bladder is unknown. The morphological classes of LUT innervation are also intriguing: anterograde labeling from DRGs found three to four types of nerve endings ranging from simple to complex, branched endings, with a single axon sometimes forming multiple types in different layers [22,23]. A unique tree-like neuron that is engaged during urination was discovered in the male rodent urethra [25]. The functions of specific morphologies and their molecular identities are unclear. Multiple functional subtypes likely exist within the broad CGRP-expressing population, for instance, and genetic tools will allow for studies connecting precise function to identity [24].

Planning when to find a restroom relies on assessing just how full the bladder is. Nerve recordings and cellular-resolution calcium imaging of DRGs revealed that distinct neurons detect innocuous and high-intensity mechanical cues in the bladder [2,26]. Individuals with loss-of-function mutations in PIEZO2, in addition to recordings from mouse models, revealed that this ion channel mediates the sensation of bladder fullness and drives efficient urinary reflexes. Interestingly, PIEZO2 is not required for high-threshold bladder stretch sensing, so this mechanism remains unknown. The related ion channel PIEZO1 might also contribute to mechanosensitivity in bladder afferents [7]. More work is needed to determine how these ion channels function in both urothelial cells and neurons to mediate different aspects of mechanical sensation, and how other mechanosensory transduction mechanisms might contribute.

The focus of this review is mechanical sensation, but chemosensation also occurs in the bladder, and some chemoreceptor neurons are polymodal, or become mechanically sensitized during inflammation [15,27]. Sensors of inflammatory mediators or irritant sensors, like TRPA1, histamine receptors, or the Mas-gene-related G protein-coupled receptor family, are expressed on bladder neurons and might cause hypersensitivity and affect the feeling of urgency or pain [27–31].

### Central circuits driving urination

#### Spinal cord and reflexes facilitating urination

Mechanical reflexes coordinate the bladder detrusor muscle and the internal and external urethral sphincters (EUSs). Stretch sensation from the bladder, conveyed through the pelvic nerve, and urethral flow and distension information, conveyed through the pudendal nerve, are important for promoting the transition from storage to voiding [32,33]. The muscle groups work together: the urethral outlet must relax during bladder contraction for efficient urination. In addition to supraspinal reflexes that drive urination, explained below, multiple spinal reflexes were defined by the physiologist Barrington, including responses to bladder distension, urethral distension, and urethral fluid flow [34]. At low volumes, stretch information from the bladder initiates a guarding reflex that enhances EUS tone to maintain continence. When a stretch threshold is reached, a supraspinal reflex initiates micturition by promoting detrusor contraction and sphincter relaxation [32]. Although not prominent in humans, rodents and dogs display bursting pattern of sphincter activity that is thought to act as an additional augmenting action or pump to promote efficient urination. A knowledge gap remains in defining the sensory subtypes that mediate these specific reflex functions, and understanding them will be critical for designing therapies to treat dysfunction in this complex, coordinated system.

The spinal cord is the first site of integration and the relay station for sensory signals that trigger micturition reflexes. Sensory neurons project into Lissauer’s tract at the apex of the dorsal horn and send collaterals laterally into lamina V-VII, and medially into lamina X at the base of the dorsal horn [16] (Figure 1). Sensory information from the bladder also projects locally to Onuf’s nucleus (dorsolateral nucleus in rodents) in the ventral horn, and these somatic motor neurons stimulate activity of the EUS to maintain continence. Interneurons in the lumbosacral spinal cord also carry sensory information to neurons at the thoracolumbar spinal level to promote sympathetic outflow through the hypogastric nerve. Sympathetic postganglionic neurons release norepinephrine (NE) to inhibit urination and maintain continence; they provide excitatory, alpha-adrenergic receptor-mediated contraction of the bladder base, dome and urethra, and inhibitory beta-adrenergic receptor-mediated relaxation of the smooth muscle in the body of the bladder. This is the target for beta3-adrenergic agonists that are used to treat overactive bladder.

#### Supraspinal micturition reflex

Although peripheral inputs facilitate and coordinate storage and voiding reflexes, it is the brainstem pontine micturition center, also called Barrington’s nucleus (Bar) that drives urination [35–37]. Before reaching the hindbrain, sacral projections first travel to the midbrain periaqueductal gray (PAG) to convey bladder filling information (Figure 1). As bladder volume increases, so does activity in the lateral and ventrolateral subdivisions of the PAG (l-vlPAG), and functional parcellation of the human PAG at 7T functional magnetic resonance imaging (fMRI), while measuring perceptions of bladder fullness, suggests the PAG as a site of integration for bladder sensory information [38]. Additionally, neurons in vlPAG project directly to Bar. Modulation of this circuitry can alter micturition bidirectionally, with GABAergic activity inhibiting urination and glutamatergic projections promptly stimulating it, which suggests that the PAG may gate the activity of Bar neurons that control micturition [37,39]. When bladder volume reaches the threshold for micturition, the switch from storage to voiding is associated with Bar activation and enhanced PAG activity [38]. Optogenetic stimulation of glutamatergic neurons in Bar promotes urination through coordinated detrusor contraction and urethral sphincter relaxation [35–37]. The nonvolitional, supraspinal micturition reflex persists following supracollicular or intercollicular decerebration (i.e. does not involve rostral or higher brain sites), and is eliminated by transections caudal to Bar, including at the level of the spinal cord [34,40,41]. Interestingly, bilateral destruction of the midbrain resulted in *‘a permanent loss of consciousness of wanting to micturate or defecate*,’ suggesting that input from rostral to this location is needed for sensation/awareness [42].

#### Barrington’s nucleus and neuronal populations

Barrington’s nucleus has multiple cell types that may contribute to its functional flexibility. Corticotropin-releasing hormone (*Crh*)-expressing glutamatergic Bar neurons have long been used as a proxy for Bar overall. We recently reported additional specific subgroups of Bar neurons, including proenkephalin (*Penk*) and *Vglut3*+/tachykinin *(Tac1)*+ populations [43], and that estrogen receptor alpha (*Esr1*) [35,44] and prolactin receptor (*Prlr*) are abundantly expressed, but not specific to one neuronal population [45]. The specific functions of the neuronal subtypes need clarification, but it is clear that activation of *Esr1*+ and also *Penk*-expressing neurons induces robust control over sphincter muscle relaxation [43] to drive efficient urination [35], and *Crh*-expressing neurons promote voiding or nonvoiding bladder contractions depending on bladder fullness [37,46]. The latter could allow the micturition control center to act as a behavioral rheostat that measures incoming sensory information to assess urgency and integrate this with external cues.

Descending projections from Bar densely innervate the intermediolateral cell column (IML) and dorsal gray commissure (DGC) region of the sacral spinal cord, with bladder motor neurons and interneurons that project to somatic EUS motor neurons in Onuf’s nucleus [47]. Parasympathetic control of the bladder detrusor muscle originates from the sacral IML, and this stimulates co-ordinated bladder contractions. Most cholinergic neurons in pelvic ganglia receive preganglionic input from the sacral spinal cord via the pelvic nerve, whereas noradrenergic pelvic neurons are innervated via the hypogastric nerve by preganglionic neurons at the thoracolumbar spinal cord level [16].

### Deciding when to go

Mammals choose carefully when and where they urinate, this reflex is under voluntary control. Because social factors also shape urination [35,36], bladder sensory information is combined with external cues about context to guide urinary behavior. Thus, Bar is not just a simple switch but receives inputs from many regions that modulate its function, and this lends insight into top-down control over interoceptive processes (Figure 1). Prominent regions with inputs to Bar include the bed nucleus of the stria terminalis (BnST), medial preoptic area (MPOA), lateral hypothalamic area (LHA), the central nucleus of the amygdala (CeA), and others [36,37]. Functional studies are beginning to reveal how these sites play a role in urinary behaviors. For example, optogenetic activation of Bar terminals of excitatory LHA neurons evokes urination, more consistently with a full bladder and with a delay of ~30 s [37], during which the mice moved to the ‘toilet’ corner of the cage. Thus, the LHA may act as a modulatory node that contributes to the decision of where and when to void, taking bladder fullness information into account. Additionally, the locus coeruleus (LC) and medial prefrontal cortex (mPFC) synchronize 20—30s before Bar activation, and this is hypothesized to mediate disengagement from ongoing behaviors to initiate voiding [48]. Mapping the network of inputs to the micturition centers in the brainstem (i.e. Bar and PAG), will provide a foundation to understand mechanisms that integrate sensory cues to make the decision to urinate.

The experience of a very full bladder without a bathroom available is unpleasant and anxiety-inducing; there is an emotional component to bladder interoception. Cortical regions that integrate interoceptive cues and affect cognitive functions are likely also involved in urinary tract sensation and decision-making. Sites like the insular cortex and anterior cingulate cortex are thought to play roles in more emotional or affective outputs, but are also central to interoception. Interestingly, functional studies show differences in insular cortex activation during bladder filling contribute to pain and pathology [49,50], and stimulation of the anterior cingulate cortex drives urination in rodents and humans [51,52]. These higher-order brain regions will be interesting to probe to investigate the affective component of urinary decisions during normal and pathological states.

#### When bladder sensation is broken

It is extremely disruptive to daily life when normal sensations that allow for planning and control over urination are impaired. The mechanics of the urinary tract are key for normal sensory function, and aging and other conditions that alter peripheral structures and innervation, such as giving birth [53], an enlarged prostate, or diabetes mellitus [54] can lead to dysfunction. Sensory issues might also contribute to bedwetting in children [55], although changes to central processing of bladder information have been observed in this condition [49]. Central brain network mechanisms may also be altered in disease states, and this could exacerbate or contribute to peripheral problems [56]. For instance, even though peripheral inputs certainly mediate pain sensation [57,58], changes in the activity of the central amygdala also contribute [50,59]. The parabrachial nucleus seems to integrate painful information from the bladder [60] and is a shared mechanism and a central hub for encoding unpleasant stimuli in multiple interoceptive and exteroceptive processes. Beyond direct physical changes or injury, psychological factors like social stress and early-life stress can also lead to urinary dysfunction. Brain regions that are altered by pain or stress and share a role in urinary interoceptive processing might influence circuit output from Bar to affect LUT function. A full understanding of circuits that detect bladder fullness and drive urination will be foundational for treating this wide array of disorders.

There are many remaining questions about mechanisms of urinary tract dysfunction in the periphery. Aging is accompanied by a number of LUT symptoms with mixed etiology. Much more work is needed to understand the molecular changes and mechanical changes that occur with aging that might exacerbate urinary dysfunction. Sacral neuromodulation is sometimes effective for treating disorders like overactive bladder and urinary retention, but precisely how this works and which neuronal subpopulations are targeted by stimulation is unclear. Clarifying these mechanisms would lead to more effective and specific therapies for different patients and disorders.

How the brain senses stretch in the bladder is known only broadly, but the field is starting to fill in specific information about the molecules, cell types, and circuits that control urination. Applying modern genetic tools with physiological and behavioral readouts, as has been done in other sensory systems, is much needed to move this field forward. Future work will offer important insight into this system and new possibilities for much-needed therapies to address the many issues that can impact the LUT. Accomplishing this is not a stretch.

## Data Availability

Data availability No data was used for the research described in the article.
